# Supplementary material for: Feedback control of Wnt signaling based on ultrastable histidine cluster co-aggregation between Naked/NKD and Axin
Source: eLife. 2020 Oct 7;9:e59879. doi: 10.7554/eLife.59879 (PMC7581431; doi:10.7554/eLife.59879)
Supplement: Figure 3—source data 1. [file elife-59879-fig3-data1.docx]

**Figure 3-source data 1**

**HisC proteins in the human genome and their subcellular location**

| **Protein** | **Localisation** | **Sequence** |
| --- | --- | --- |
| ERC2 | cytoplasm | HHHYHHHHHHHHH |
| Axin1 | cytoplasm | HHHRHVHHHVHH |
| SORBS2 | cytoplasm | HHHHHHHHHHHRH |
| CBL | cytoplasm | HHHHHHHLSPH |
| IQSEC2 | cytoplasm | HHHHHHHHHGHSH |
| USP34 | cytoplasm | HHHHHHHHHHHHDGH |
| NKD2 | cytoplasm | HEHHHHHEHHHHHHHHHFH |
| NKD1 | cytoplasm | HEHHHHHEHHHHYHH |
| SYNGAP1 | cytoplasm | HHHHHHHHHH |
| RHOBTB2 | cytoplasm | HPEDHQGHSDQHHHHHHHHH |
| EPB41L4B | cytoplasm | HHHQHQHQHQHQHH |
| SHANK1 | cytoplasm | HHHPPHHHHHHAPPPQPHHHHAH |
| DLGAP3 | membrane | HTSHHHHHHHHHHHHQSRH |
| CPEB4 | membrane | HHPHHPHFQHHHSQH |
| BEAN1 | membrane | HRHRHHRHHHHHHHH |
| SIAH3 | mitochondrion | HPHHLSHHHCHHRHHHHLRHHAHPHHLHH |
| CACNA1A | membrane | HRQHHHHHHHHHH |
| CACNA1G | membrane | HHLVHHHHHHHHHYH |
| CACNA1H | membrane | HTASVHHLVYHHHHHHHHHYHFSH |
| SLC39A6 | membrane | HHHHDYHHILHHHHHQNHHPHSH; HIHHDHDHHSDHEHHSDHERHSDHEHHSEHEHHSDHDHHSHHNH; HLLPHSHASHHHSHSH |
| PRRT1 | membrane | HHHHHHHYH |
| VGLL3 | nuclear | HHHPHAHMHHRHRHHHHHHH |
| CBX4 | nuclear | HPPSHHPHPHPHHHHHHHHHHHH |
| CCNT1 | nuclear | HKEKHKTHPSNHHHHHNHHSHKHSH |
| MAF | nuclear | HHHHHHAAGHHHH |
| ONECUT2 | nuclear | HHPHPHHHPHHHHHHHH |
| HAND1 | nuclear | HHHHHHHPHPAHPMLH |
| POU3F3 | nuclear | HHHHHHHHAHPHPPHPHHAQGPPHH |
| YY1 | nuclear | HGHAGHHHHHHHHHHH |
| OTX1 | nuclear | HHHHHPHAHHPLSQSSGHHHHHHHHHH |
| HOXA1 | nuclear | HHHHHHHHHH |
| MEOX2 | nuclear | HHRGHHHHHHHHHHHHH |
| FOXG1 | nuclear | HHASHGHHNSHHPQHHHHHHHHHHH |
| POU4F1 | nuclear | HHHHHHHHH |
| POU4F2 | nuclear | HHHHHHHHHHHHQPH |
| FOXF2 | nuclear | HAHPHHHHHHHVPH |
| DYRK1A | nuclear | HHHHGNSSHHHHHHHHHHHHH |
| FAM76B | nuclear | HHPKHHHHHHHHHHRHSSSHH |
| FOXB2 | nuclear | HLHPHHHHHPHHHHHHHAAAHHHHHHH |
| TAF2 | nuclear | HSDHHHHHHHEH |
| VGLL2 | nuclear | HGHLHQGATEPWHHAHPHHAHPHH |
| ARID1B | nuclear | HQQHHHHHHAHHHHHHAHHLHHHH |
| MAFA | nuclear | HHHGAHHAAHHHHAAHHHHHHHHHH |
| AUTS2 | nuclear | HDYSHHHHHHHH |
| NR4A3 | nuclear | HHHHHHHHHHHHHHQQQH |
| GATA6 | nuclear | HHHHHHHHHH |
| CDX2 | nuclear | HPHHHPHHHPHH |
| FOXC2 | nuclear | HHHQHHGHHH |
| GSX2 | nuclear | HAHHHHHPPQHHHHHH |
| PRDM13 | nuclear | HHHHHHAHHHHH |
| CHD8 | nuclear | HHHHHHPHPHHHHHHH |
| BMP2K | nuclear | HHHHHHHH |
| FAM120C | nuclear | HHHPAHHFHHH |
| ONECUT1 | nuclear | HHHHHHHHHHHPHHH |
| ZNF281 | nuclear | HHHHHHHH |
| MAFB | nuclear | HHHHHHHHPHPHHA; HAHPHHHHHH |
| ZIC3 | nuclear/cytoplasm | HHHHHHHHHHH |
| ZIC2 | nuclear/cytoplasm | HHHHHHHHH |
| TSC22D1 | nuclear/cytoplasm | HPHHLHHHHQIHHGHHLQHGHHH |
| SKOR2 | nuclear/cytoplasm | HSAQTHPHHHHHPHHHHHHHH |
| RNF111 | nuclear/cytoplasm | HGHHFQHHHHHHHTPH |
| NUFIP2 | nuclear/cytoplasm | HHHSHHHPHHHPQQQQQQPHHHHHYYFYNHSHNHHHHHHH |
| NLK | nuclear/cytoplasm | HHHHHHHHLPHLPPPHLHHHHHPQHHLH |
| FOXA2 | nuclear/cytoplasm | HHHSHHHHQPH |
| NCAN | secreted | HRMRRHHHHHQHHHQHHHH |
| HRG | secreted | HPHKHHSHEQHPHGHHPHAHHPHEHDTHRQHPHGHHPHGHHPHGHHPHGHHPHGHHPHCH |
| SEPP1 | secreted | HYHHEHHHNHGHQH |
| BTBD11 | membrane | HHHHHHHALH |
| HRCT1 | membrane | HHHRHPGHVSHVPNVGLHHHHHPRHTPHHLHHHHHPHRHHPRH |
| PRICKLE3 | cytoplasm | HHHHNHHHHHNRH |
| C21orf58 | nuclear | HHHHHHHH |
| SKIDA1 | nuclear/cytoplasm | HHHHHHHHHHHHHHHRAQPPQQSHHPPHHH |
| RBM33 | nuclear/cytoplasm | HPPQHQHHHHHHH |
| LRCH1 | cytoplasm | HPLHHPHHHHHHHQHH; HAHPHHHHHH |
