# Supplementary material for: Feedback control of Wnt signaling based on ultrastable histidine cluster co-aggregation between Naked/NKD and Axin
Source: eLife. 2020 Oct 7;9:e59879. doi: 10.7554/eLife.59879 (PMC7581431; doi:10.7554/eLife.59879)
Supplement: Figure 5—source data 1. [file elife-59879-fig5-data1.docx]

**Figure 5-source data 1**

**Oligonucleotides used for CRISPR engineering of HEK293T cells and confirmation of lesions**

| oligonucleotide | sequence |
| --- | --- |
| *H. Sapiens* NKD1 forward primer exon6 | 5’-CAAATTGCCGCTCCTTTGCC-3’ |
| *H. Sapiens* NKD1 reverse primer exon6 | 3’-CCCTAGAGCTCTGGGACTGT-5’ |
| *H. Sapiens* NKD1 sequencing primer exon6 | 5’-GAAGACCCACAGACCACCAG-3’ |
| *H. Sapiens* NKD2 forward primer exon6 | 5’-GCCAGTGTGTCCTCTTCTCC-3’ |
| *H. Sapiens* NKD2 reverse primer exon6 | 5’-GCCATCCTTGCATCCACCAA-3’ |
| *H. Sapiens* NKD2 sequencing primer exon6 | 5’-CTTCTCCTTCTGGGTGACGT-3’ |
| *H. Sapiens* NKD1 forward primer exon10 | 5’-GAAGCCATCCACATCCCACA -3’ |
| *H. Sapiens* NKD1 reverse primer exon10 | 3’-AGGCCACTCAAAACCAGAGT-5’ |
| *H. Sapiens* NKD1 sequencing primer exon10 | 5’-CCAGAGGGGCAAGAAACAAG-3’ |
| *H. Sapiens* NKD2 forward primer exon10 | 5’-AAGCAGTTCCTCAAGTCCCC-3’ |
| *H. Sapiens* NKD2 reverse primer exon10 | 3’-AGATGTGTTCACCGCCATGT-5’ |
| *H. Sapiens* NKD2 sequencing primer exon10 | 3’-CCTACAGCAGTCACCCCTTG-5’ |
