# Supplementary material for: Feedback control of Wnt signaling based on ultrastable histidine cluster co-aggregation between Naked/NKD and Axin
Source: eLife. 2020 Oct 7;9:e59879. doi: 10.7554/eLife.59879 (PMC7581431; doi:10.7554/eLife.59879)
Supplement: Figure 6—source data 1. [file elife-59879-fig6-data1.docx]

**Figure 6-source data 1**

**Oligonucleotides used for Drosophila CRISPR engineering and confirmation of lesions**

| oligonucleotide | sequence |
| --- | --- |
| *D. melanogaster nkd* forward primer exon2 | 5’-AGCATCCCACCCCTCTCTTC-3’ |
| *D. melanogaster nkd* reverse primer exon2 | 3’-TGGGTGCGGAAGATGGAATC-5’ |
| *D. melanogaster nkd* sequencing primer exon2 | 5’-TCCGAGGAGCTGATGTACCA-3’ |
| *D. melanogaster nkd* forward primer exon5 | 5’-TGGAGGCCTCTCTCTTTCAA-3’ |
| *D. melanogaster nkd* reverse primer exon5 | 3’-GCATCGCCGTAGATCTTGGA-5’ |
| *D. melanogaster nkd* sequencing primer exon5 | 5’-CCCAAGCCAATGTGAGTCCC-3’ |
