# Supplementary material for: Feedback control of Wnt signaling based on ultrastable histidine cluster co-aggregation between Naked/NKD and Axin
Source: eLife. 2020 Oct 7;9:e59879. doi: 10.7554/eLife.59879 (PMC7581431; doi:10.7554/eLife.59879)
Supplement: Figure 8—source data 1. [file elife-59879-fig8-data1.docx]

**Figure 8-source data 1**

**Full list of BioID hits obtained with wt or mutant Nkd1-BirA***

| Protein | GFP | wt | 4A | G2A | ΔHisC | Protein | GFP | wt | 4A | G2A | ΔHisC |
| --- | --- | --- | --- | --- | --- | --- | --- | --- | --- | --- | --- |
| ACACA | 36 | 534 | 433 | 526 | 387 | TFRC | 1 | 11 | 11 | 1 | 1 |
| ZC3HAV1 | 14 | 316 | 271 | 22 | 266 | FAM135A | 1 | 10 | 13 | 1 | 18 |
| DVL2 | 29 | 178 | 105 | 303 | 157 | GAB1 | 1 | 10 | 10 | 1 | 48 |
| ACACB | 6 | 173 | 121 | 156 | 88 | JPH1 | 2 | 10 | 8 | 1 | 1 |
| DVL3 | 24 | 160 | 91 | 240 | 146 | KIAA0430 | 1 | 10 | 8 | 10 | 1 |
| APC | 1 | 157 | 54 | 20 | 125 | TMEM201 | 1 | 10 | 1 | 1 | 4 |
| ZFYVE16 | 15 | 142 | 120 | 4 | 101 | TRIM41 | 1 | 10 | 8 | 2 | 1 |
| ERBB2IP | 18 | 129 | 73 | 8 | 375 | APPL1 | 1 | 9 | 8 | 1 | 1 |
| TTF1 | 17 | 125 | 129 | 1 | 1 | AXIN1 | 1 | 9 | 4 | 1 | 2 |
| FLJ26213 | 14 | 103 | 68 | 3 | 128 | CEP89 | 1 | 9 | 4 | 1 | 24 |
| N4BP1 | 9 | 102 | 102 | 22 | 11 | FLJ37871 | 1 | 9 | 9 | 1 | 1 |
| DVL1 | 8 | 93 | 46 | 127 | 69 | FLJ52077 | 1 | 9 | 4 | 1 | 1 |
| EPHA2 | 1 | 90 | 86 | 12 | 73 | FLJ76245 | 1 | 9 | 15 | 1 | 34 |
| FLJ61530 | 4 | 90 | 67 | 1 | 13 | PCDH7 | 1 | 9 | 1 | 1 | 20 |
| CPD | 10 | 85 | 71 | 1 | 85 | PTPRG | 1 | 9 | 2 | 1 | 26 |
| NKD1 | 1 | 84 | 91 | 130 | 230 | ARHGAP32 | 1 | 8 | 3 | 1 | 14 |
| FLJ78684 | 4 | 70 | 63 | 27 | 3 | CXorf58 | 1 | 8 | 6 | 1 | 1 |
| KIAA0323 | 1 | 64 | 42 | 8 | 15 | FLJ13167 | 1 | 8 | 5 | 5 | 1 |
| SCRIB | 7 | 59 | 55 | 11 | 287 | FLJ53115 | 1 | 8 | 5 | 1 | 1 |
| FLJ75601 | 1 | 56 | 58 | 24 | 1 | FLJ54361 | 1 | 8 | 12 | 1 | 14 |
| DLG5 | 1 | 54 | 51 | 32 | 134 | FLJ77258 | 1 | 8 | 7 | 2 | 15 |
| KEAP1 | 5 | 52 | 43 | 53 | 6 | KIAA1549 | 1 | 8 | 6 | 1 | 16 |
| SQSTM1 | 2 | 47 | 67 | 23 | 1 | MPZL1 | 1 | 8 | 3 | 1 | 17 |
| ITSN1 | 7 | 43 | 32 | 1 | 24 | SERPINB12 | 1 | 8 | 9 | 2 | 4 |
| STAM | 7 | 42 | 30 | 1 | 1 | SLC12A2 | 1 | 8 | 3 | 1 | 45 |
| VANGL1 | 2 | 41 | 36 | 7 | 46 | TAX1BP1 | 1 | 8 | 1 | 1 | 1 |
| FLJ77107 | 2 | 35 | 27 | 3 | 34 | ATXN3 | 1 | 7 | 6 | 1 | 1 |
| TRIM26 | 2 | 35 | 38 | 29 | 20 | EFNB1 | 1 | 7 | 5 | 1 | 24 |
| ANKRD26 | 3 | 33 | 24 | 15 | 153 | ELAVL1 | 1 | 7 | 5 | 3 | 2 |
| ASCC3 | 1 | 30 | 23 | 18 | 2 | FLJ14522 | 1 | 7 | 2 | 1 | 15 |
| TNKS | 1 | 30 | 13 | 23 | 28 | KIF26B | 1 | 7 | 5 | 1 | 11 |
| ROR2 | 1 | 29 | 26 | 2 | 37 | PPFIBP1 | 1 | 7 | 10 | 1 | 33 |
| ZFYVE9 | 1 | 29 | 16 | 1 | 5 | SHISA2 | 1 | 7 | 1 | 1 | 3 |
| LSR | 1 | 27 | 23 | 1 | 54 | SLC38A1 | 1 | 7 | 7 | 1 | 31 |
| ROBO1 | 4 | 26 | 10 | 5 | 45 | STON2 | 1 | 7 | 5 | 1 | 9 |
| KIRREL | 1 | 25 | 21 | 1 | 22 | TRAFD1 | 1 | 7 | 8 | 2 | 1 |
| MARK3 | 1 | 25 | 13 | 2 | 78 | AMER1 | 1 | 6 | 5 | 1 | 1 |
| OCLN | 1 | 25 | 15 | 2 | 41 | ARL13B | 1 | 6 | 4 | 1 | 11 |
| FLT4 | 4 | 23 | 27 | 1 | 1 | ATP1A1 | 1 | 6 | 1 | 1 | 9 |
| LRP6 | 1 | 23 | 13 | 1 | 1 | CDC42BPA | 1 | 6 | 3 | 1 | 34 |
| PKP4 | 1 | 23 | 18 | 1 | 29 | CUL3 | 1 | 6 | 2 | 1 | 1 |
| ROR1 | 1 | 22 | 21 | 1 | 14 | EFR3A | 1 | 6 | 2 | 1 | 4 |
| TMEM2 | 1 | 22 | 11 | 2 | 33 | FAM171A1 | 1 | 6 | 9 | 1 | 20 |
| DSG2 | 2 | 21 | 24 | 1 | 67 | FARP2 | 1 | 6 | 1 | 1 | 7 |
| FLJ76779 | 1 | 21 | 8 | 1 | 1 | FLG2 | 1 | 6 | 3 | 1 | 1 |
| ITGB1 | 2 | 21 | 9 | 1 | 43 | FLJ12728 | 1 | 6 | 8 | 1 | 22 |
| NOTCH2 | 1 | 21 | 14 | 1 | 66 | FLJ54710 | 1 | 6 | 3 | 1 | 1 |
| PIK3C2B | 1 | 20 | 8 | 1 | 6 | FLJ61343 | 1 | 6 | 5 | 3 | 2 |
| ROBO2 | 1 | 19 | 10 | 1 | 34 | FLJ75725 | 1 | 6 | 3 | 1 | 1 |
| PTPN23 | 1 | 18 | 9 | 1 | 1 | FLJ76780 | 1 | 6 | 4 | 1 | 26 |
| CACHD1 | 1 | 17 | 6 | 1 | 23 | FLJ78619 | 1 | 6 | 1 | 1 | 11 |
| KIAA1217 | 1 | 17 | 5 | 1 | 43 | GSDMA | 1 | 6 | 8 | 3 | 2 |
| SDK2 | 1 | 17 | 5 | 1 | 16 | GTPBP4 | 1 | 6 | 11 | 12 | 6 |
| Megalin | 1 | 16 | 5 | 1 | 1 | HSP90B1 | 1 | 6 | 11 | 1 | 1 |
| PARD3 | 3 | 16 | 14 | 4 | 97 | KIAA1109 | 1 | 6 | 1 | 1 | 8 |
| RPS6KC1 | 1 | 16 | 9 | 1 | 9 | N4BP2 | 1 | 6 | 14 | 1 | 1 |
| SDK1 | 1 | 16 | 8 | 1 | 23 | ATP2B1 | 1 | 6 | 1 | 1 | 1 |
| AKAP12 | 2 | 15 | 7 | 1 | 38 | PLCH1 | 1 | 6 | 2 | 2 | 31 |
| CXADR | 1 | 15 | 14 | 2 | 47 | PRKDC | 1 | 6 | 6 | 4 | 6 |
| IBTK | 1 | 15 | 16 | 12 | 15 | STX6 | 1 | 6 | 3 | 1 | 15 |
| LZTS2 | 2 | 15 | 15 | 3 | 28 | TIAM1 | 1 | 6 | 1 | 1 | 14 |
| CELSR1 | 1 | 14 | 2 | 1 | 3 | TRIM13 | 1 | 6 | 3 | 1 | 14 |
| SNX29 | 1 | 14 | 11 | 1 | 8 | UNC5B | 1 | 6 | 7 | 1 | 17 |
| CUEDC1 | 1 | 13 | 11 | 4 | 2 | USP54 | 1 | 6 | 6 | 9 | 8 |
| FLOT2 | 1 | 13 | 8 | 1 | 33 | USP6NL | 1 | 6 | 2 | 1 | 15 |
| INADL | 1 | 13 | 8 | 5 | 44 | ZC3HDC1 | 1 | 6 | 1 | 1 | 1 |
| KIF14 | 2 | 13 | 11 | 21 | 20 | ARG1 | 1 | 5 | 2 | 2 | 1 |
| PDZD4 | 1 | 13 | 2 | 1 | 1 | ARHGAP22 | 1 | 5 | 5 | 1 | 14 |
| PKP2 | 1 | 13 | 18 | 4 | 9 | CCDC77 | 1 | 5 | 1 | 13 | 4 |
| TOM1L2 | 1 | 13 | 6 | 3 | 4 | CTNNB1 | 1 | 5 | 2 | 1 | 11 |
| CCDC8 | 1 | 12 | 4 | 1 | 17 | DEPDC1B | 1 | 5 | 1 | 1 | 16 |
| FCHO2 | 1 | 12 | 4 | 1 | 13 | DLG3 | 1 | 5 | 2 | 1 | 4 |
| FLJ75689 | 2 | 12 | 9 | 1 | 1 | FLJ34950 | 1 | 5 | 2 | 8 | 5 |
| HIP1 | 1 | 12 | 13 | 8 | 1 | FLJ42130 | 1 | 5 | 7 | 6 | 2 |
| KIAA0754 | 1 | 12 | 7 | 1 | 64 | FLJ77041 | 1 | 5 | 1 | 1 | 1 |
| KIAA0922 | 1 | 12 | 14 | 1 | 1 | FLJ78647 | 1 | 5 | 2 | 1 | 2 |
| MARK1 | 1 | 12 | 6 | 1 | 1 | FRS2 | 1 | 5 | 3 | 1 | 26 |
| RABEP1 | 1 | 12 | 13 | 1 | 1 | HRNR | 1 | 5 | 1 | 1 | 1 |
| UCH | 1 | 12 | 6 | 1 | 3 | IPO8 | 1 | 5 | 11 | 7 | 5 |
| WDR51B | 1 | 12 | 23 | 23 | 12 | PARP14 | 1 | 5 | 1 | 1 | 1 |
| YTHDC2 | 2 | 12 | 13 | 27 | 8 | PCDH19 | 1 | 5 | 5 | 1 | 6 |
| DSG1 | 1 | 11 | 7 | 1 | 1 | PPP1R16B | 1 | 5 | 1 | 1 | 6 |
| LMTK2 | 1 | 11 | 6 | 1 | 1 | RELL1 | 1 | 5 | 1 | 1 | 9 |
| PHLDB2 | 1 | 11 | 1 | 1 | 41 |  |  |  |  |  |  |
