# Supplementary material for: Feedback control of Wnt signaling based on ultrastable histidine cluster co-aggregation between Naked/NKD and Axin
Source: eLife. 2020 Oct 7;9:e59879. doi: 10.7554/eLife.59879 (PMC7581431; doi:10.7554/eLife.59879)
Supplement: Supplementary file 1. [file elife-59879-supp1.pdf]

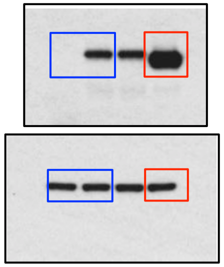

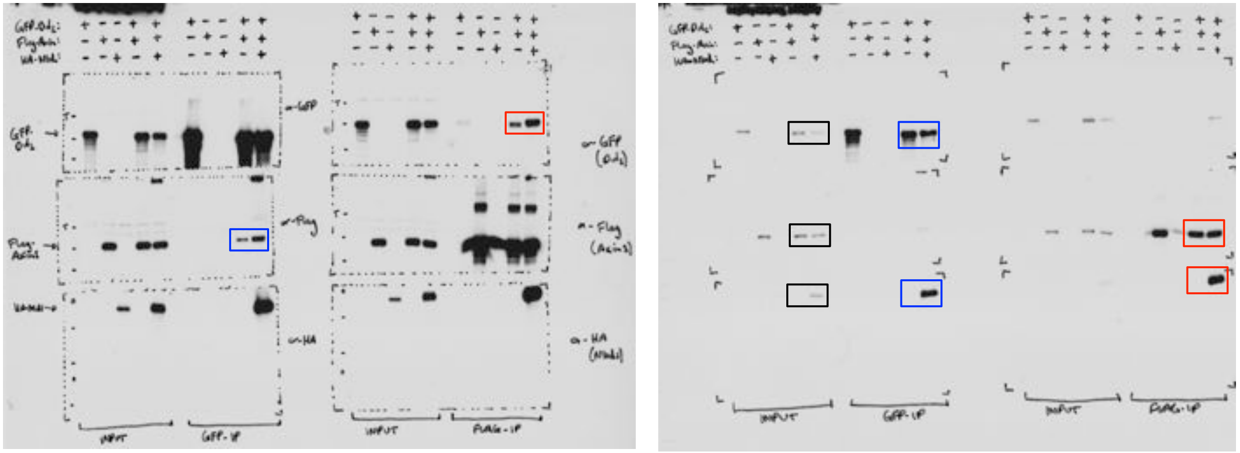

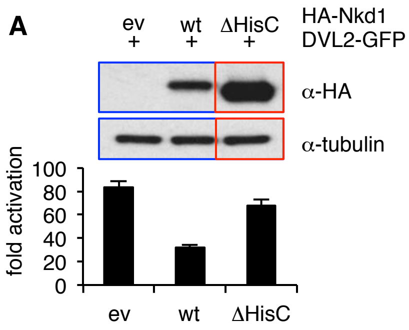

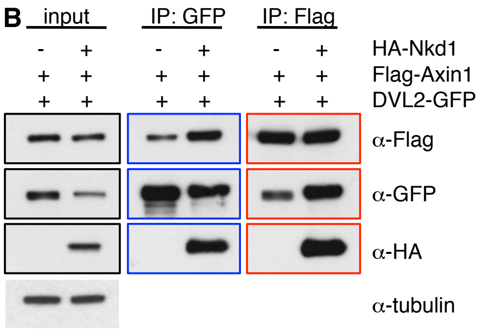

Figure 1 Gammons et al.

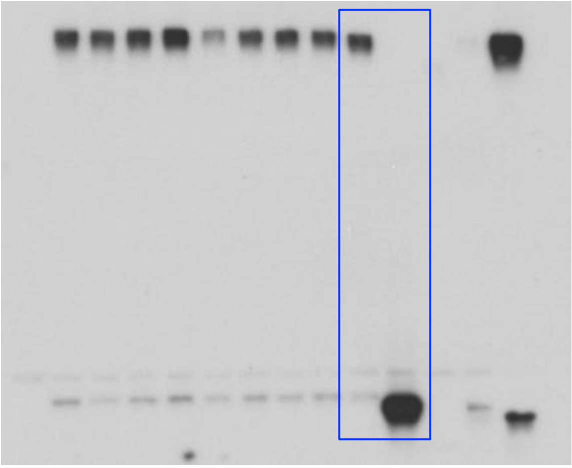

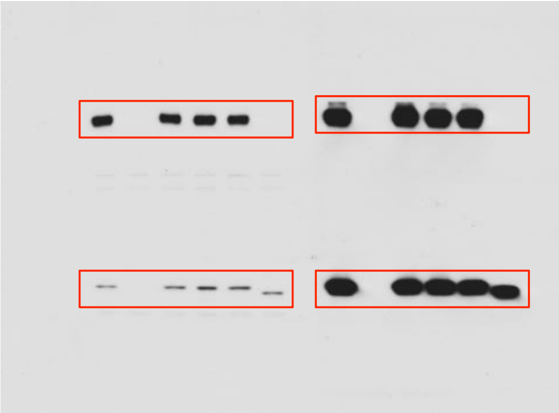

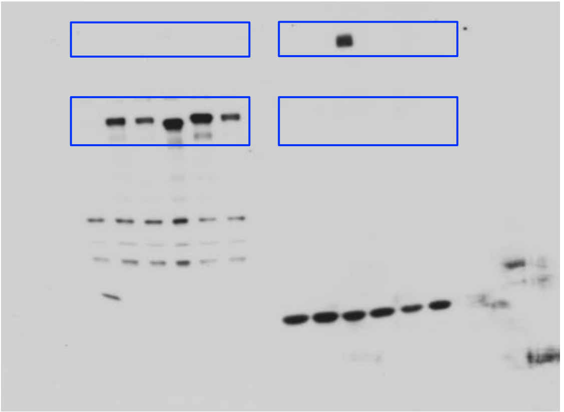

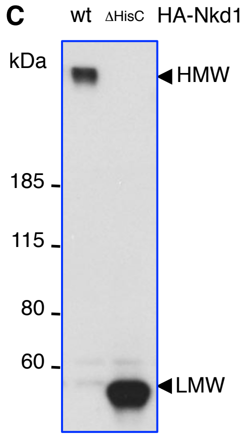

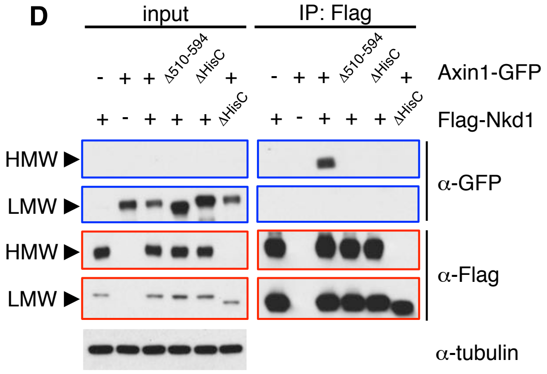

Figure 1 Gammons et al.

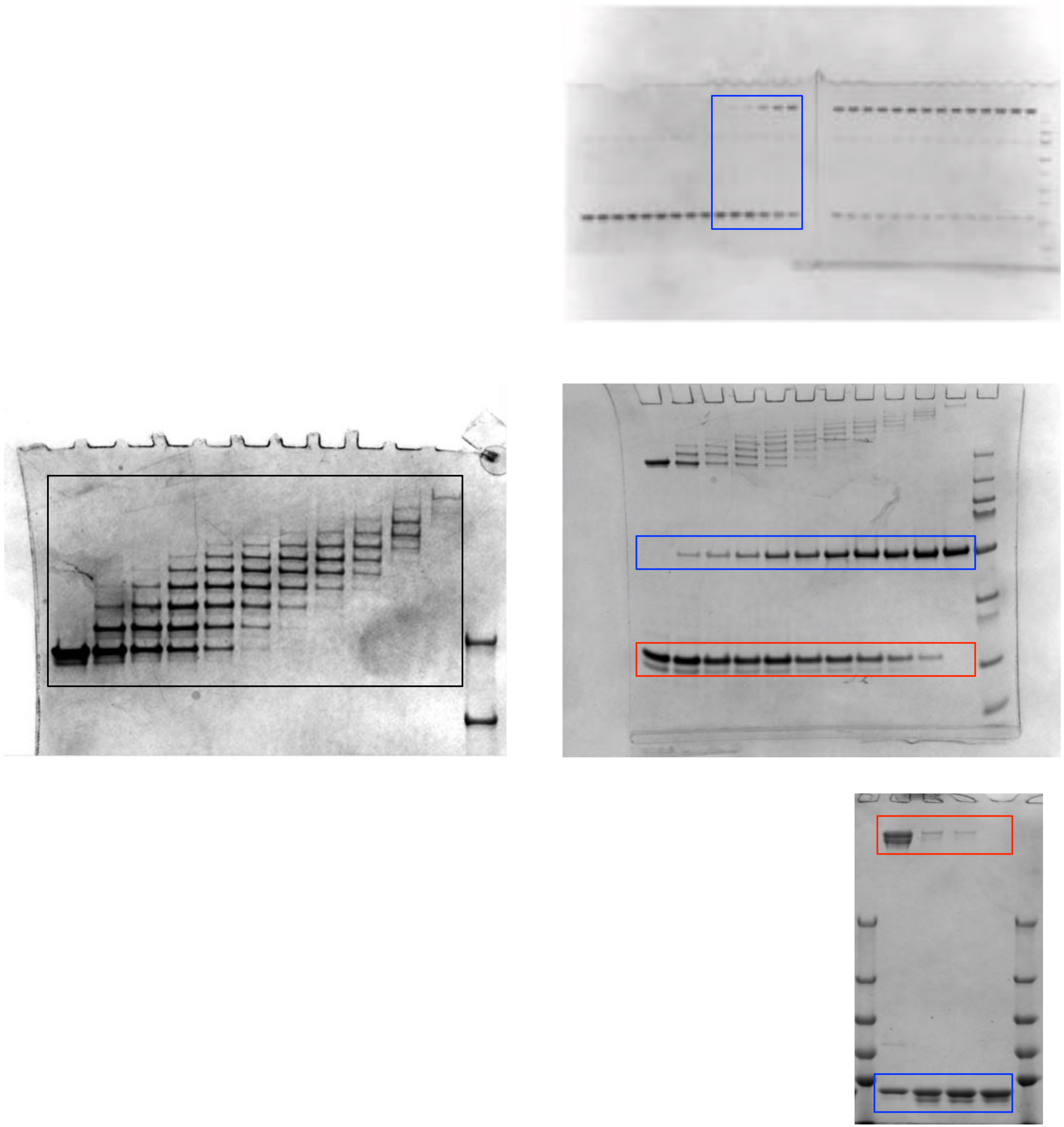

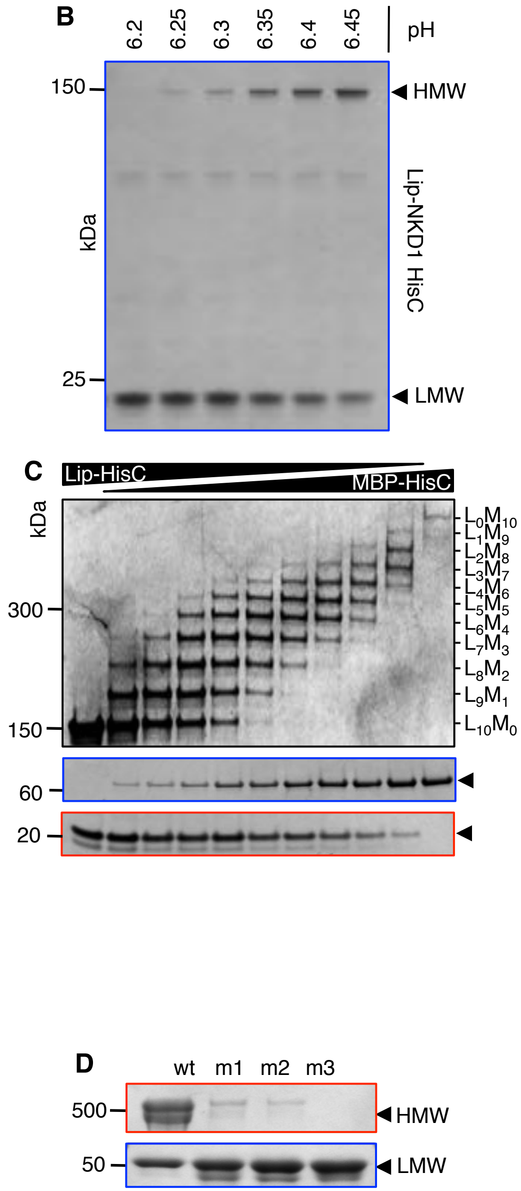

Figure 2 Gammons et al.

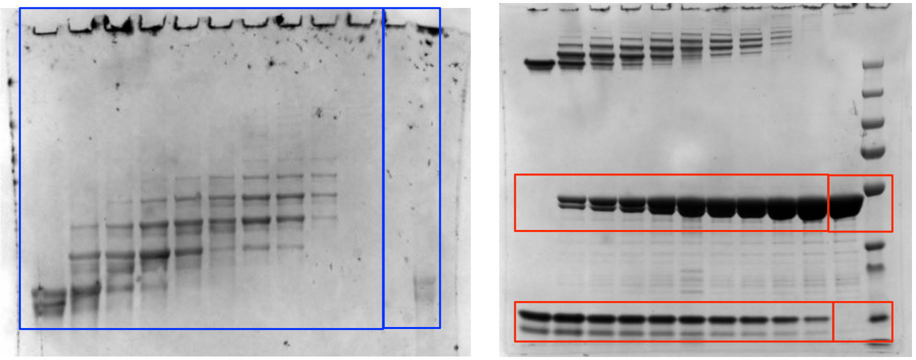

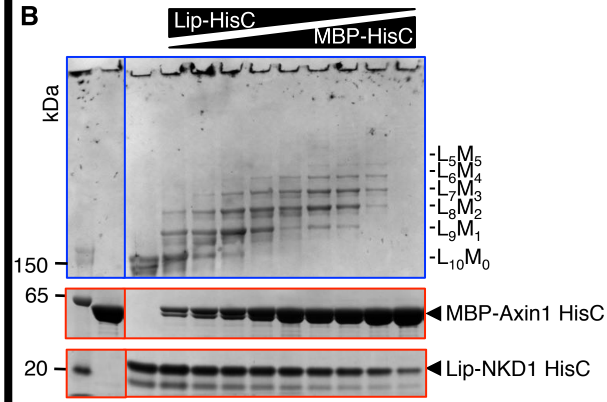

Figure 3 Gammons et al.

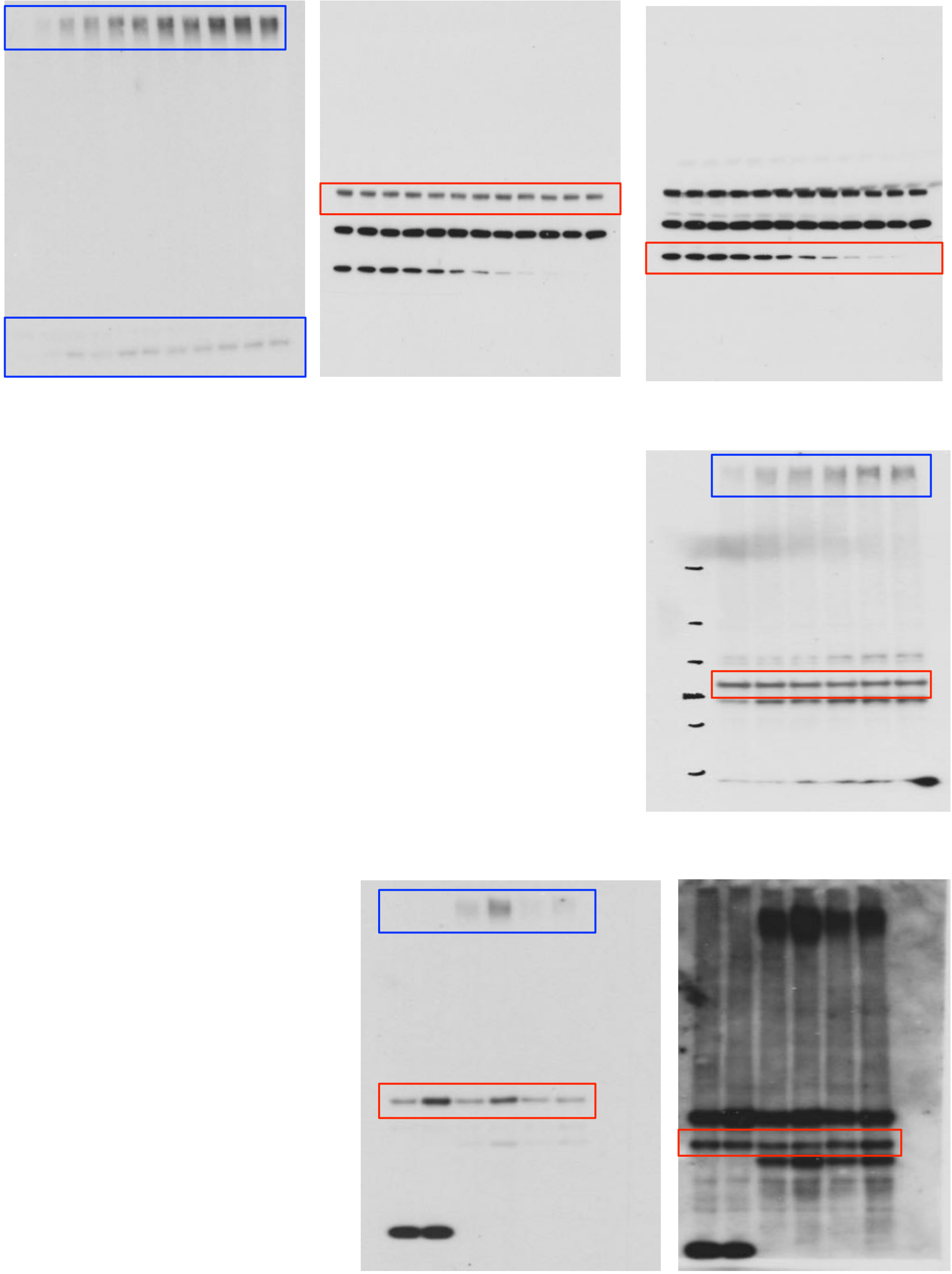

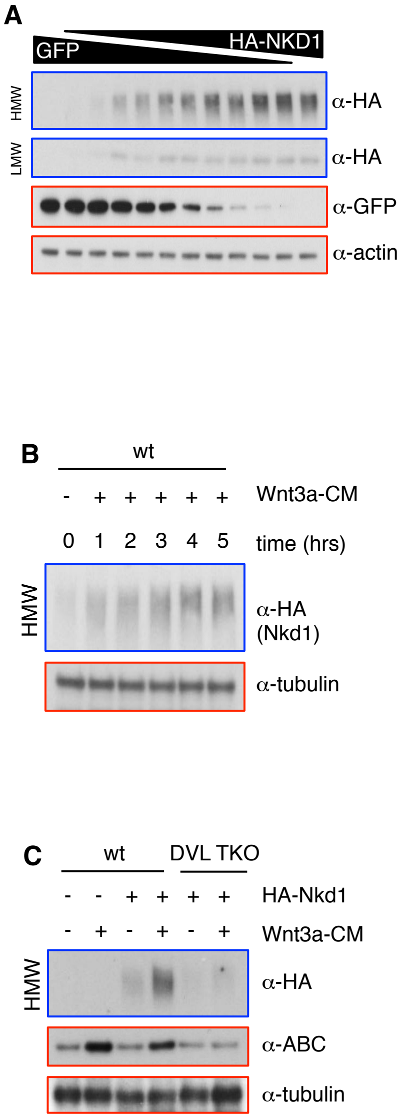

Figure 4 Gammons et al.

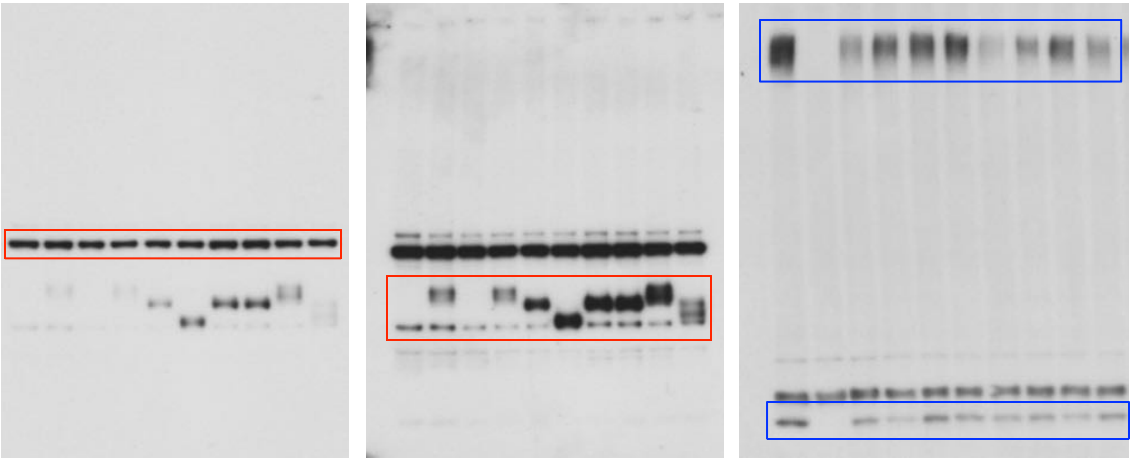

D

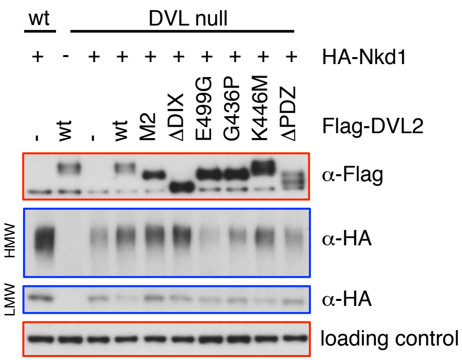

Figure 4 Gammons et al.

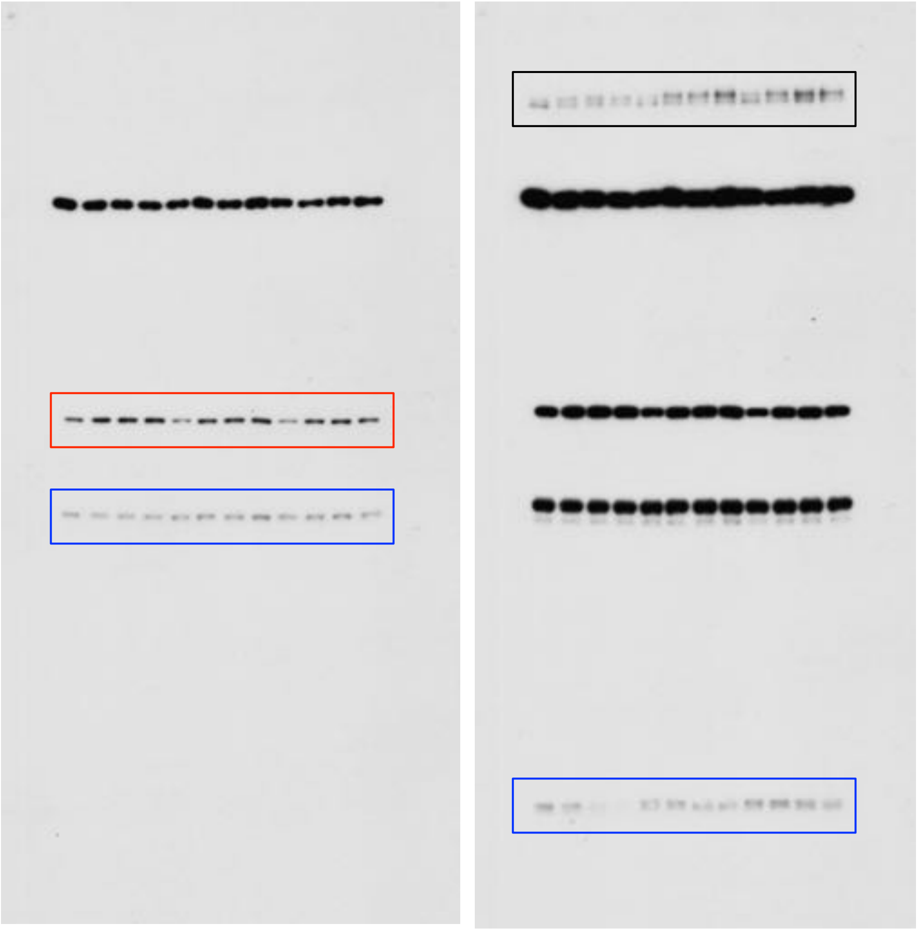

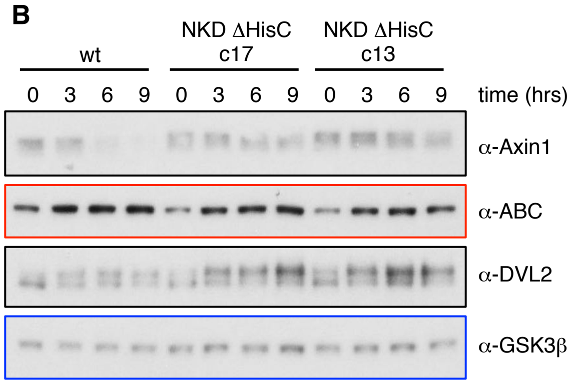

Figure 5 Gammons et al.

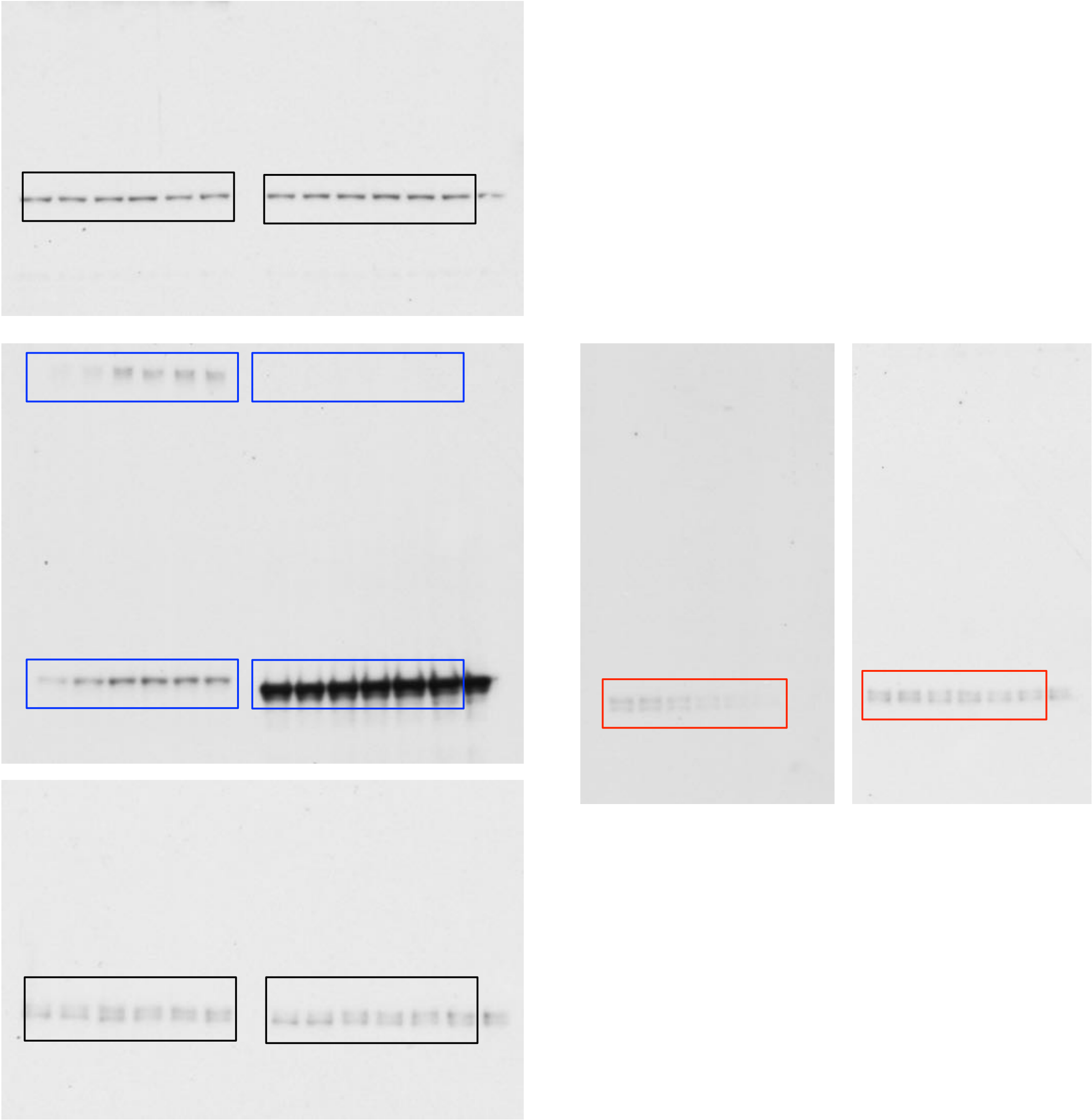

C

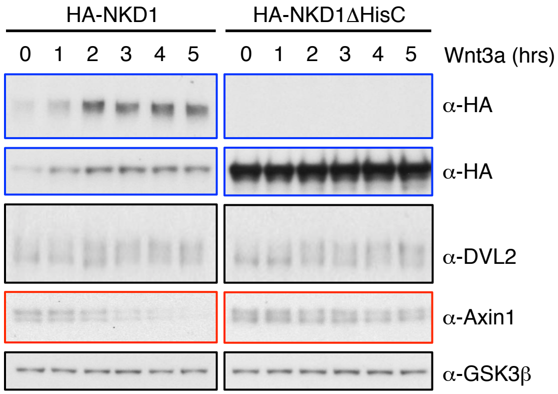

Figure 5 Gammons et al.

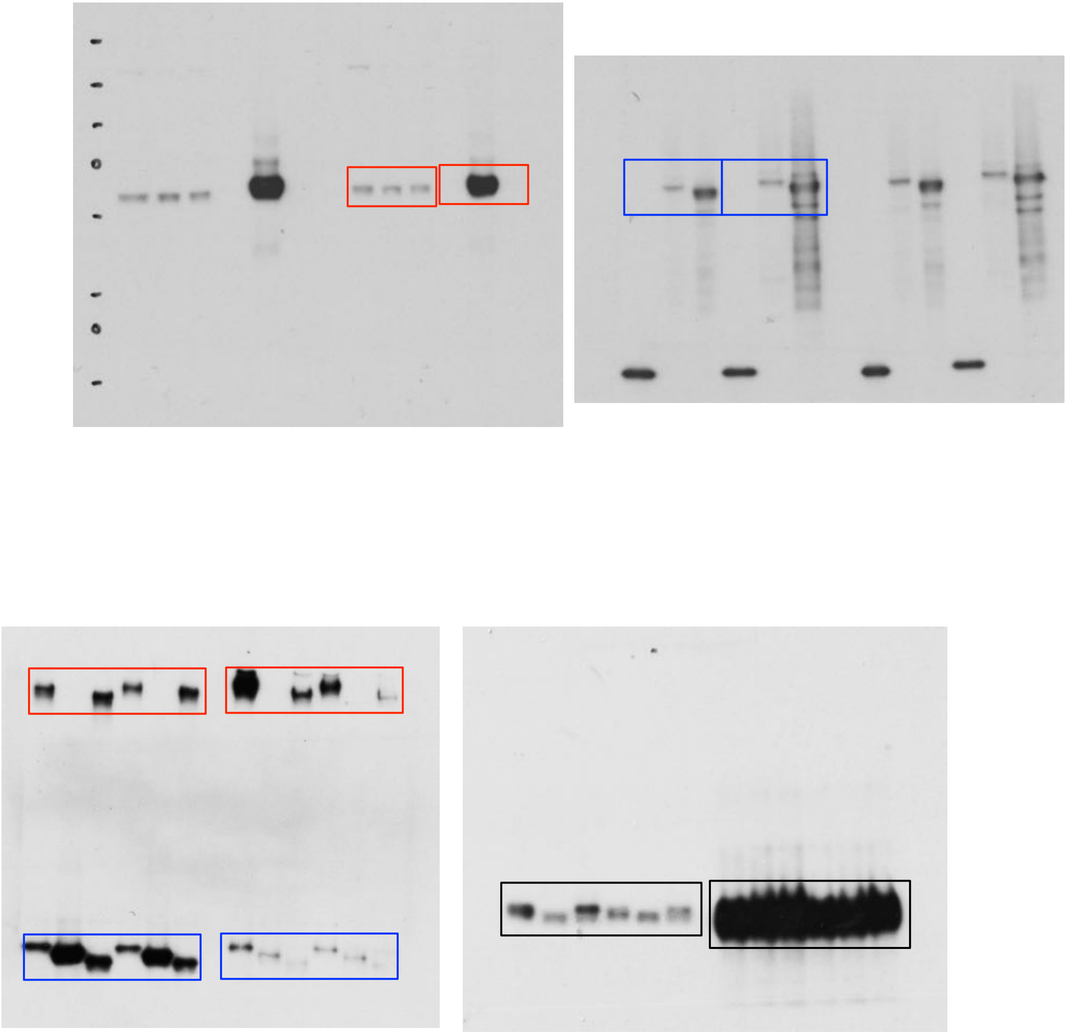

C

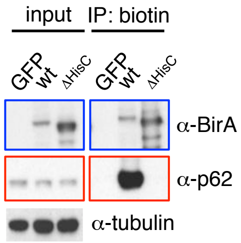

D

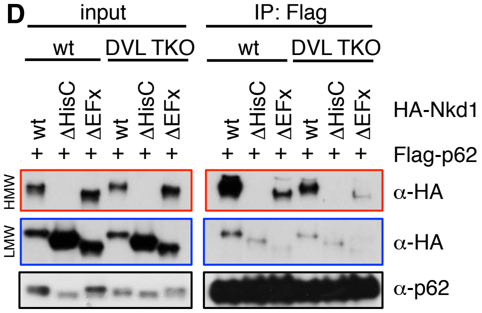

Figure 8 Gammons et al.

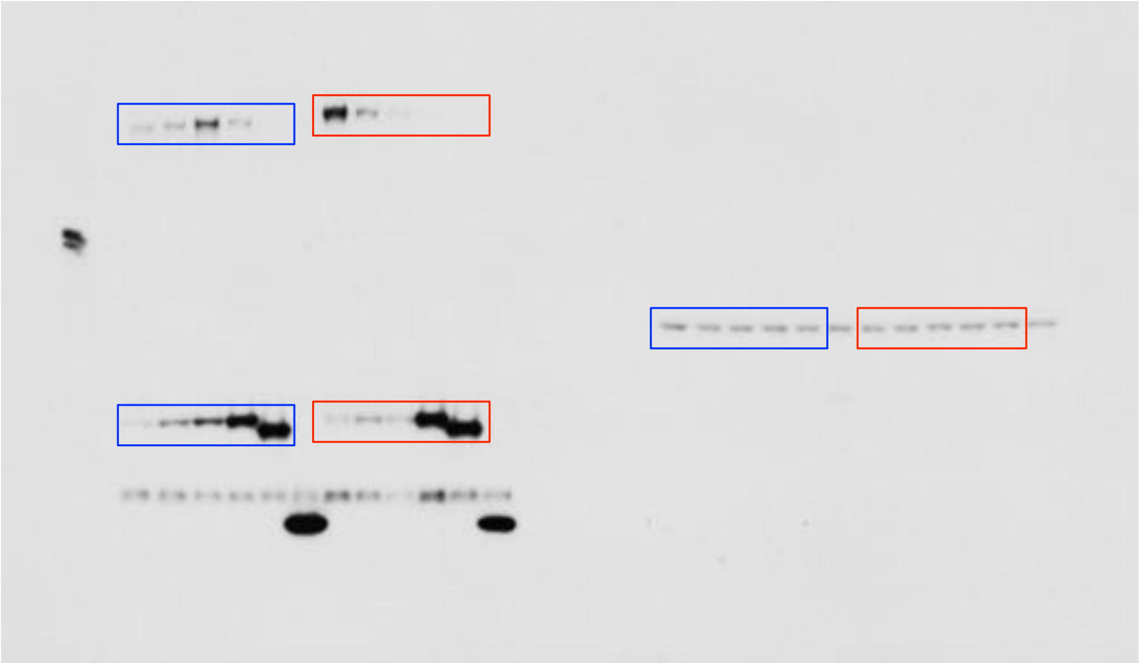

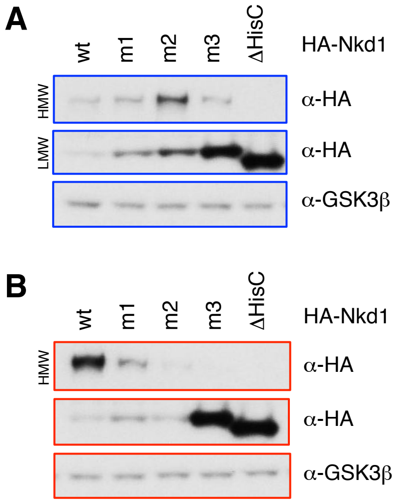

Figure S3 Gammons et al.

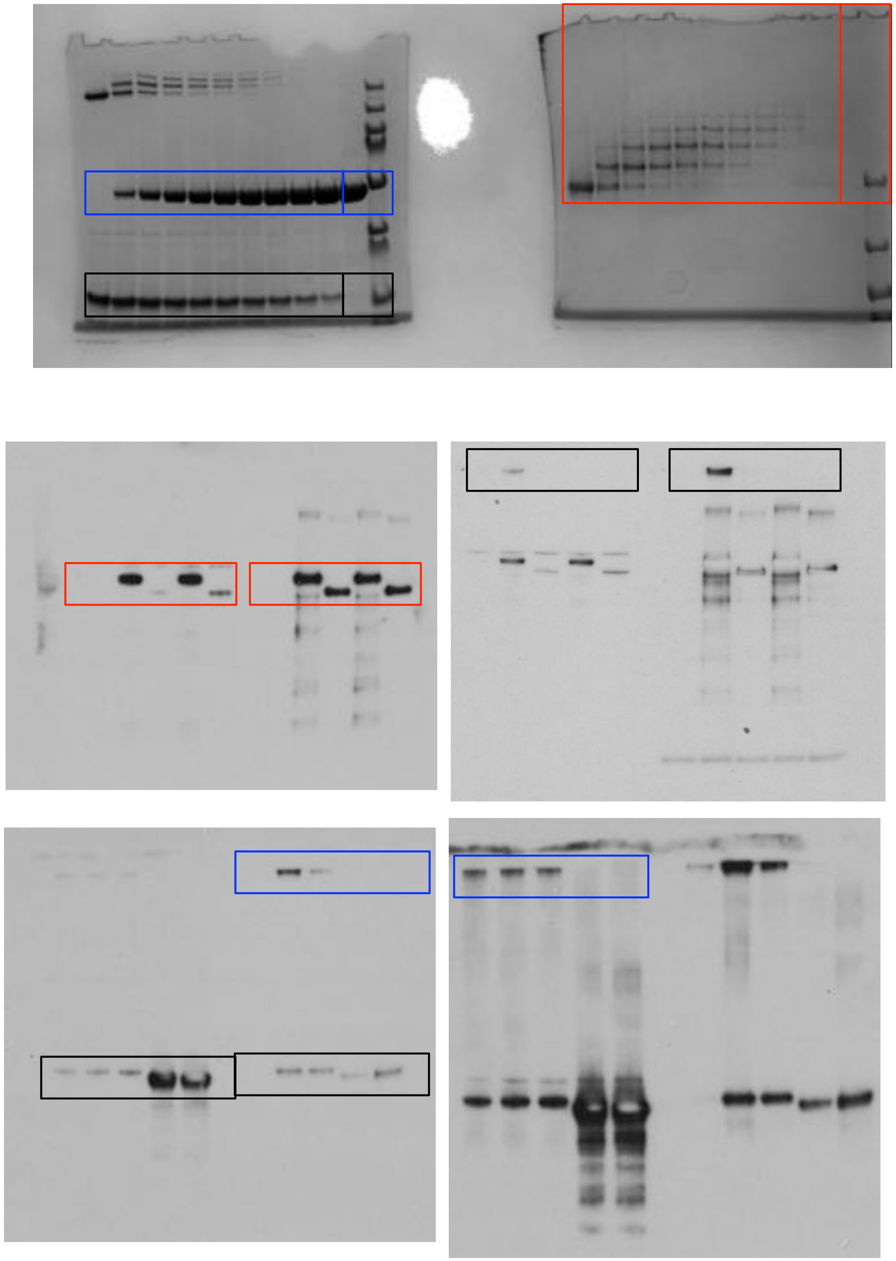

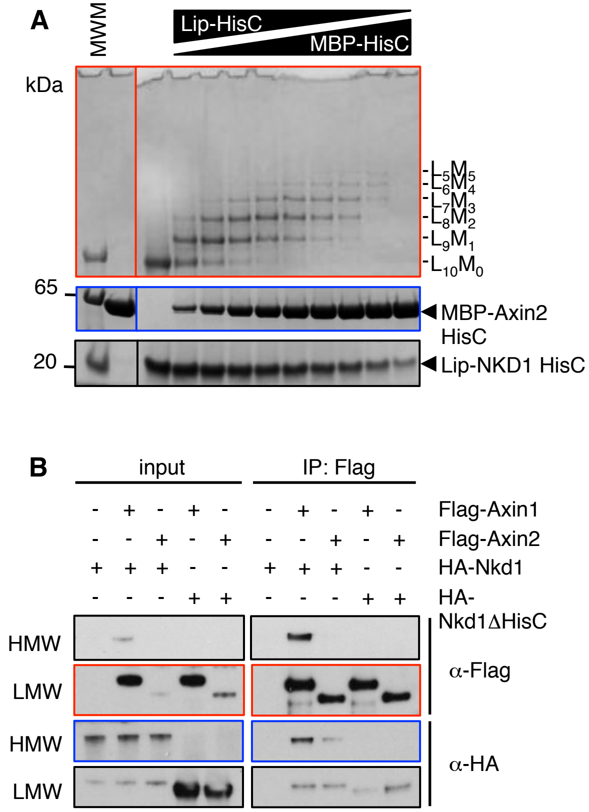

Figure S4 Gammons et al.

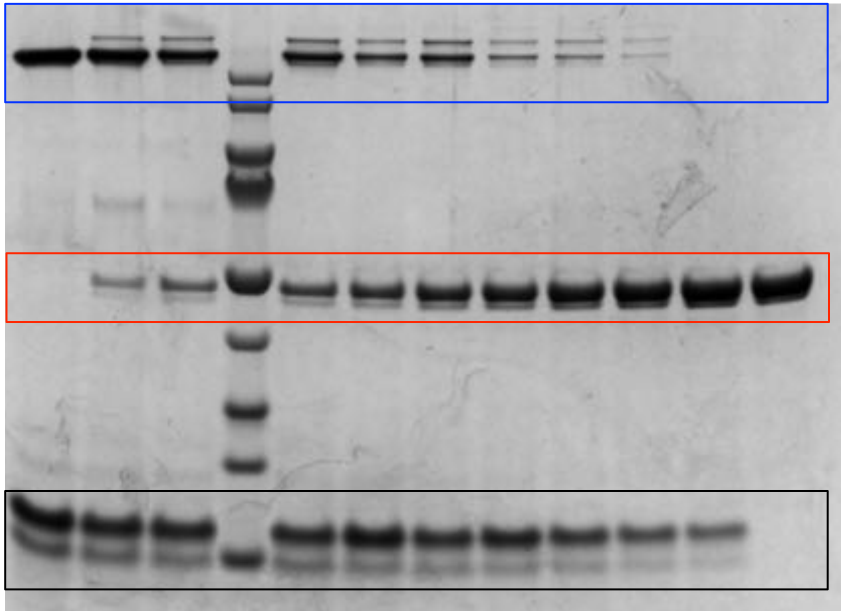

Example: all others gels cropped in the same way

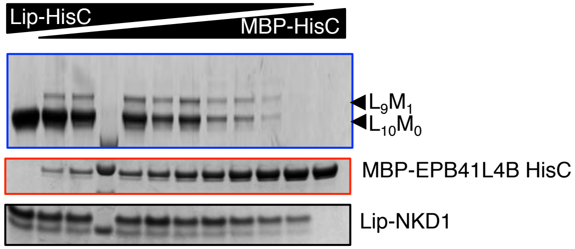

Figure S5 Gammons et al.

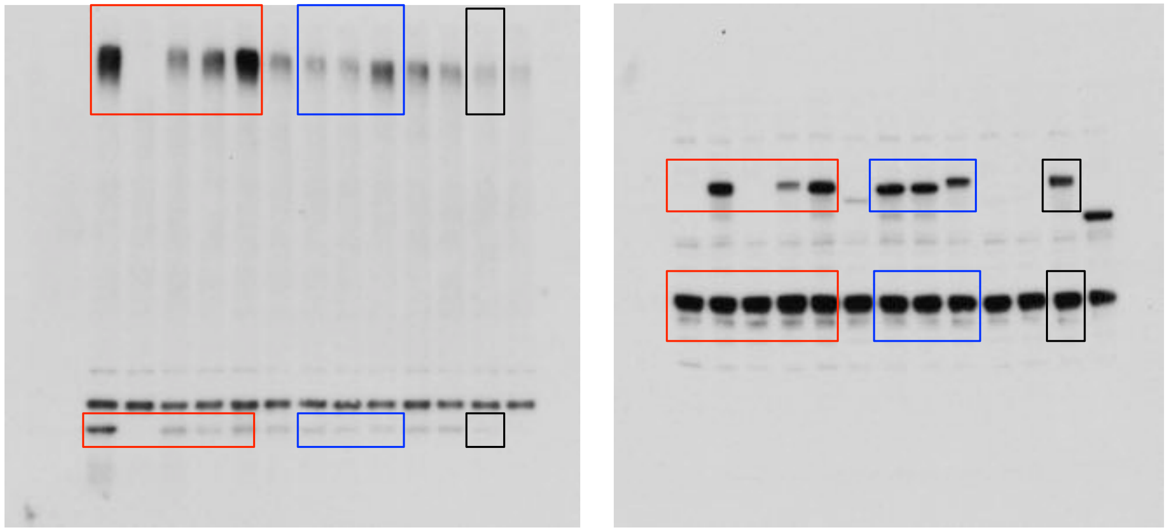

B

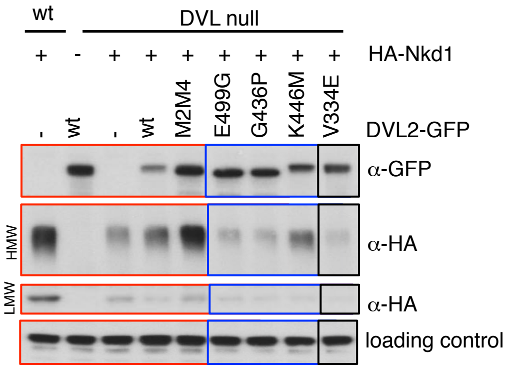

Figure S6 Gammons et al.

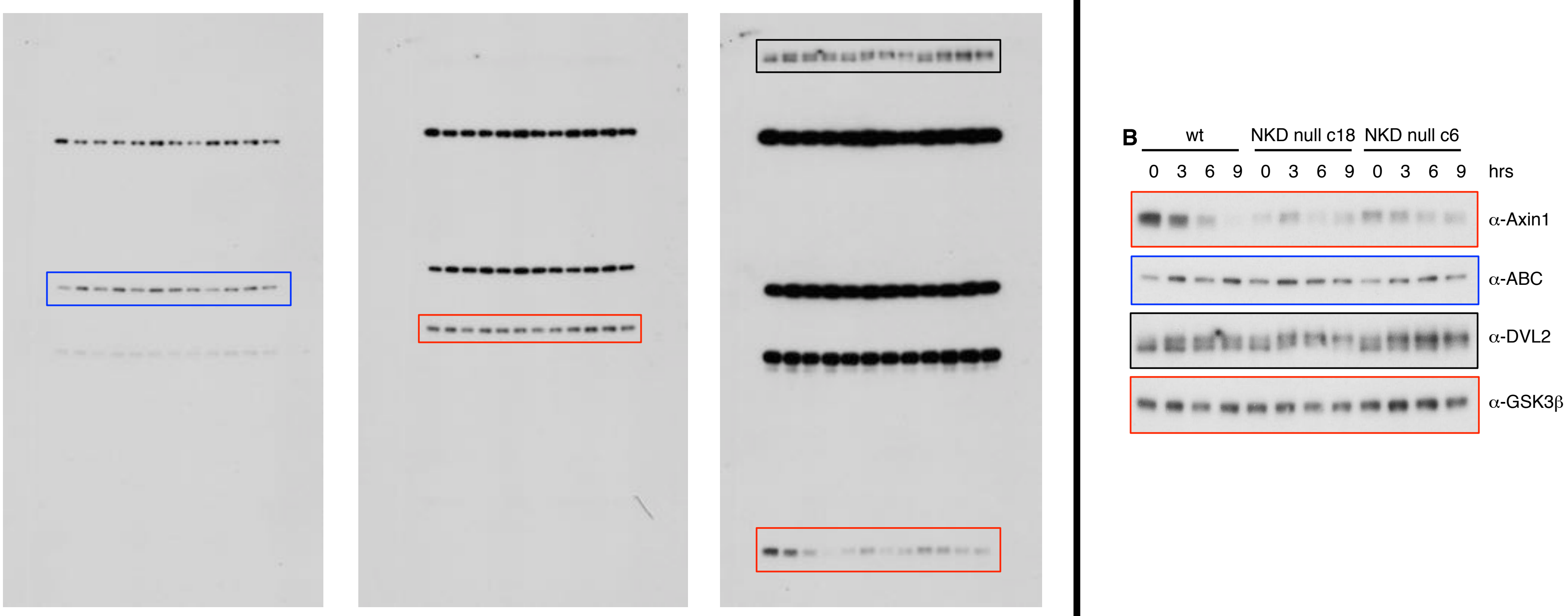

Figure S8 Gammons et al.
